# Machine learning identification of thresholds to discriminate osteoarthritis and rheumatoid arthritis synovial inflammation

**DOI:** 10.1186/s13075-023-03008-8

**Published:** 2023-03-02

**Authors:** Bella Mehta, Susan Goodman, Edward DiCarlo, Deanna Jannat-Khah, J. Alex B. Gibbons, Miguel Otero, Laura Donlin, Tania Pannellini, William H. Robinson, Peter Sculco, Mark Figgie, Jose Rodriguez, Jessica M. Kirschmann, James Thompson, David Slater, Damon Frezza, Zhenxing Xu, Fei Wang, Dana E. Orange

**Affiliations:** 1 Hospital for Special Surgery, 535 E 70th Street, New York, NY 10009 USA; 2 Weill Cornell Medicine, New York, NY USA; 3 Columbia University Vagelos College of Physicians and Surgeons, New York, NY USA; 4 Stanford University, Stanford, CA USA; 5 The MITRE Corporation, McLean, VA USA; 6 The Rockefeller University, New York, NY USA

**Keywords:** Osteoarthritis, Rheumatoid arthritis, Synovial inflammation, Histology, Machine learning

## Abstract

**Background:**

We sought to identify features that distinguish osteoarthritis (OA) and rheumatoid arthritis (RA) hematoxylin and eosin (H&E)-stained synovial tissue samples.

**Methods:**

We compared fourteen pathologist-scored histology features and computer vision-quantified cell density (147 OA and 60 RA patients) in H&E-stained synovial tissue samples from total knee replacement (TKR) explants. A random forest model was trained using disease state (OA vs RA) as a classifier and histology features and/or computer vision-quantified cell density as inputs.

**Results:**

Synovium from OA patients had increased mast cells and fibrosis (*p* < 0.001), while synovium from RA patients exhibited increased lymphocytic inflammation, lining hyperplasia, neutrophils, detritus, plasma cells, binucleate plasma cells, sub-lining giant cells, fibrin (all *p* < 0.001), Russell bodies (*p* = 0.019), and synovial lining giant cells (*p* = 0.003). Fourteen pathologist-scored features allowed for discrimination between OA and RA, producing a micro-averaged area under the receiver operating curve (micro-AUC) of 0.85±0.06. This discriminatory ability was comparable to that of computer vision cell density alone (micro-AUC = 0.87±0.04). Combining the pathologist scores with the cell density metric improved the discriminatory power of the model (micro-AUC = 0.92±0.06). The optimal cell density threshold to distinguish OA from RA synovium was 3400 cells/mm^2^, which yielded a sensitivity of 0.82 and specificity of 0.82.

**Conclusions:**

H&E-stained images of TKR explant synovium can be correctly classified as OA or RA in 82% of samples. Cell density greater than 3400 cells/mm^2^ and the presence of mast cells and fibrosis are the most important features for making this distinction.

**Supplementary Information:**

The online version contains supplementary material available at 10.1186/s13075-023-03008-8.

## Background

Joint damage in the knee can be severe in both osteoarthritis (OA) and rheumatoid arthritis (RA) such that total knee replacement (TKR) is often the only management option [1]. More than 700,000 TKRs are performed annually in the USA, and explanted tissue is often stained with hematoxylin and eosin (H&E) and evaluated by a pathologist as the standard of care. The physical exam of the knees of patients with OA can be similar to that of patients with RA, that is, both conditions can be characterized by joint swelling, warmth, and effusion. Pathology reports regarding the extent of synovial inflammation can be another useful piece of information for the managing clinician to discriminate ongoing RA-related disease activity from coincident primary OA in patients with longstanding RA. Therefore, establishing a precise level of synovial tissue inflammation for future investigators could provide a fast, inexpensive, and clinically meaningful benchmark for patient assessment.

Several investigators have sought to optimize methods to score synovial inflammation using H&E-stained synovial tissue samples to distinguish OA from RA. For example, Krenn et al. [2–4] developed a widely cited scoring algorithm that includes semi-quantitative assessments of three synovial features identifiable on H&E-stained synovium: inflammatory infiltrates, lining hyperplasia, and stromal activation, a measure of cellularity that encompasses fibroblasts, endothelial cells, and giant cells. It is challenging to distinguish macrophages from fibroblasts in H&E-stained images, and as a result, some groups have modified the Krenn scoring system, adopting assessments of inflammatory infiltrates and lining hyperplasia, but not stromal activation, to score synovitis [5]. In an effort to further improve sensitivity and specificity, assessments of five immunohistochemistry-stained features (CD31, CD3, CD68, CD20, and Ki67) were recently added to the Krenn score [6]. Since immunohistochemistry is not as widely available and is more expensive than H&E, our group has been studying whether assessing additional histological features in H&E-stained sections, such as plasma cells, Russell bodies, binucleate plasma cells, neutrophils, mast cells, and lining and sub-lining giant cells as well as extracellular features such as fibrin, detritus, fibrosis, and mucoid degeneration, might be useful for discriminating various types of synovial inflammation. We previously reported that plasma cells, binucleate plasma cells, Russell bodies, fibrin, neutrophils, and synovial lining giant cells were predictive of high inflammatory gene expression subsets in RA [7].

Another challenge in using semi-quantitative assessments of synovitis is the disagreement between human pathologist scores of the same sample due to the subjective grading of synovial features. Since synovial inflammation tends to be patchy, it is likely that one source of inter- and intra-rater variability is that human pathologists make a subjective choice to assess certain high-power fields in any given whole slide image. Automated computer vision quantification of cell density on whole slide images removes the requirement for subjective selection of a certain field of interest, is reproducible, is scalable as it does not require the technical expertise of a pathologist, and captures granular information about the number of cells in a synovial sample, which is very onerous to manually count by a pathologist. We previously developed and validated a computer vision algorithm to automatically count each cell nucleus in an H&E-stained synovial whole slide image in 170 RA patient synovial samples [8]. This algorithm uses classical computer vision techniques to identify synovial tissue and nuclei and yields a value of cell density, as identified by mean stained nuclei count per mm^2^ of tissue. Using this approach, we found that mean whole slide image synovial cell density in RA is strongly correlated with human pathologist scores and bulk tissue RNA-seq gene expression inflammatory subset. We hypothesized that the computer vision quantification of cell density in addition to human pathologist scores would be useful in discriminating OA from RA. Here, we employed machine learning to calculate optimal thresholds to discriminate OA from RA-related synovial inflammation using human pathologist scores of fourteen histology features as well as computer vision quantification of mean cell density in a cohort of 147 OA patients and 60 RA patients undergoing knee arthroplasty.

## Methods

### Study design and cohort

We compared knee synovial histologic features from two different cohorts of patients undergoing TKR for OA or RA at a high-volume, tertiary care hospital. This was a secondary analysis of OA and RA patients that were identified via electronic medical records or physician referral and enrolled during their preoperative screening visit.

The OA patients were enrolled in the OA subtypes cohort from November 2018 through October 2019. Patients over the age of 45 that met ACR Clinical/Radiographic Criteria, ACR Clinical/Laboratory Criteria [9], or Kellgren-Lawrence (KL) Radiographic Criteria (grades 2–4) for knee OA [9, 10] were included in the study. Patients who had a fracture in the operative knee, a diagnosis of a systemic rheumatic disease such as RA, or any disease other than OA as an indication for TKR were excluded from the study. In addition, three patients were excluded from the study sample after TKR because the pathologist assessment of the arthroplasty explant revealed a rheumatic disease diagnosis masked as OA.

As previously described, RA patients were enrolled in the RA Perioperative FLARE Study from October 2013 to October 2021 [7, 11, 12]. Inclusion criteria for this cohort were patients above the age of 18 who met the American College of Rheumatology (ACR)/European League Against Rheumatism 2010 classification criteria for RA [13] and/or the ACR 1987 criteria for RA [14]. Patients who had any other systemic rheumatic disease or crystalline arthropathy were excluded.

Written informed consent was obtained for all participants. Patients meeting the inclusion/exclusion criteria were enrolled in the respective OA and RA cohorts. Demographic characteristics such as patient age, race, sex, and body mass index (BMI) were collected. Erythrocyte sedimentation rate (ESR), C-reactive protein (CRP), rheumatoid factor (RF), and cyclic citrullinated peptide (CCP) were measured on all OA and RA patients. RF and CCP were measured as part of the standard of care in RA patients, or if unavailable, were performed by serum ELISA as in OA patients.

As per institutional policy, ethical approval for this study was provided by the Institutional Review Board at the Hospital for Special Surgery (IRB #2018-0895 and #2014-233), and the research was performed in accordance with the relevant guidelines and regulations. The study methods and results are described in accordance with the Strengthening of Reporting in Observational studies in Epidemiology (STROBE) guidelines for cohort studies [15].

### Tissue processing and histologic scoring

Synovial samples were obtained intra-operatively from 147 OA patients and 60 RA patients. As per the study protocol, orthopedic surgeons were requested to preferentially obtain a research sample from grossly abnormal-looking synovium. Tissue for histological examination was chosen by a pathologist on the basis of gross features including the smoothness and granularity of the synovial surface, red or brown discoloration, and the clarity, dullness, or opacity of the synovial layer, preferentially avoiding regions of electro-cautery effect.

Synovial samples were preferentially obtained from the most grossly inflamed (dull and opaque) area of the synovium. If there was no obviously inflamed synovium, samples were obtained from standard locations: the femoral aspects of the medial and lateral gutters and the central supratrochlear region of the suprapatellar pouch. OA synovial tissue samples were formalin-fixed and paraffin-embedded, and the RA tissues were fresh-frozen in optimal cutting temperature compound. Each tissue biopsy was sectioned at 5-μm thickness and stained with Harris-modified hematoxylin solution and eosin Y (H&E) manufactured by Epredia in Kalamazoo, MI. An expert musculoskeletal pathologist (ED) scored fourteen synovial histologic features in a single section for each patient: lymphocytic inflammation, mucoid change, fibrosis, fibrin, germinal centers, lining hyperplasia, neutrophils, detritus, plasma cells, binucleated plasma cells, Russell bodies, sub-lining giant cells, synovial lining giant cells, and mast cells. Detailed methods for scoring these features are included in the Appendix, some of which are described in prior studies [8] and available at www.hss.edu/pathology-synovitis.

### Computer vision analysis of cell density

**Fig. 1:**
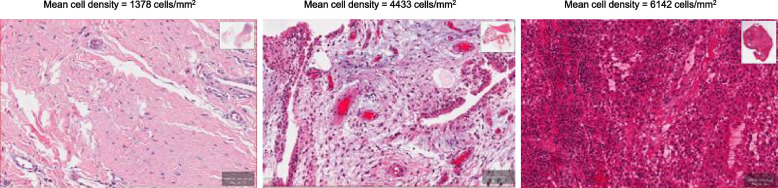
Representative images of varying nuclei densities

Pathology slides were digitized using an Aperio AT Turbo Scanner manufactured by Leica Biosystems in Deer Park, IL, USA, with a 20× resolution of the whole slide image. As previously described [8], we applied computer vision techniques on the whole slide images to count the cell nuclei and quantify the amount of tissue present. The whole slide images were deconstructed into smaller image tiles, each covering an area of approximately 0.25 mm^2^. These tiles were transformed into grayscale, analyzed for different intensity levels, and assigned a metric based on the proportion of the tile determined to contain tissue. Using a combination of techniques—including Otsu’s method [16], the watershed algorithm, and local adaptive thresholding—the cell nuclei were isolated from the tissue within the image. Final nuclei counts were refined using shape filtering and nuclei density was calculated by normalizing the total count of individual nuclei by the tissue area. This method yields a continuous value of mean cell count per mm^2^ of tissue. Pre-processing the whole slide image into tiles takes an average of 40 min, which enables the computation of nuclei density in under a minute. The open-access code can be downloaded here: https://github.com/sgmitre/ai-histology. See Fig. 1 for representative histological images of varying nuclei densities.

### Data analysis

Demographic characteristics of the OA and RA patients are reported as frequencies, means, standard deviations (SD), medians, and interquartile ranges (IQR). Chi-square tests were used to compare fourteen pathologist-graded histology scores between OA and RA patients. Logistic regression models were performed to distinguish OA vs RA as the outcome and adjusting for fibrosis and mast cell scores with lymphocytic infiltrates.

### Supervised machine learning analysis

**Fig. 2:**
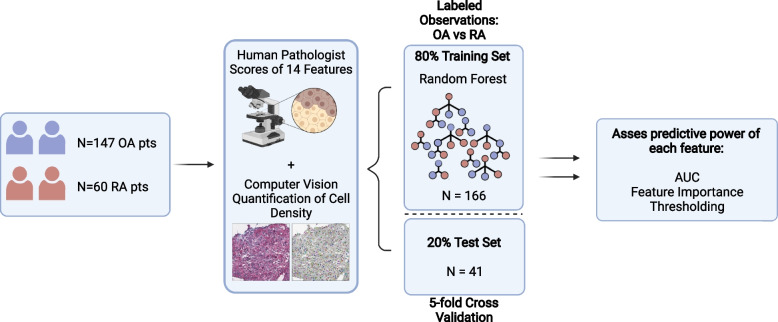
Overview of the analysis pipeline. OA osteoarthritis, RA rheumatoid arthritis, AUC area under receiver operating characteristic curves. Created with BioRender.com

A supervised machine learning model was built to classify OA vs RA samples using Random forests (Fig. 2). The model inputs were either all fourteen pathologist scores, the computer vision score alone, or both sets of scores combined. The model is selected according to the area under the receiver operating curve (AUC). The hyperparameters of the random forest model we tuned include the number of trees and the depth of each tree, which were optimized with a nested 5-fold cross-validation process (5-fold for the outer loop and 5-fold for the inner loop) [17] from candidate values [10, 20, 30, 40, 50, 60, 70, 80, 90, 100, 110, 120, 130, 140, 150, 160, 170, 180, 190, 200] and [5, 6, 8, 10, 12, 14, 16, 18, 20], respectively. The outer loop separates the data into 5 equal folds with stratified partition. For each iteration, one specific fold will be used as a testing and the rest 4 folds as training. Then another 5-fold cross-validation procedure will be performed on the training set to estimate the optimal model hyperparameters. The final results were reported using macro-AUC and micro-AUC on the testing data. For micro-AUC, we computed the AUC of each fold and reported the average AUC and standard deviation (SD). For macro-AUC, we concatenated the AUC from all folds of the testing data [18]. Such a nested cross-validation process can help obtain a robust estimation on the model’s generalization performance [17].

Additionally, to determine the discriminative power of each individual pathology feature in distinguishing OA vs RA, we treated the feature values themselves as prediction scores for generating the receiver operating characteristic (ROC) curve, based on which the AUC value was calculated. Then, to determine the optimal threshold for a given feature to distinguish OA vs RA, Youden’s *J* statistic was calculated to obtain the optimal point on the ROC curve, the optimal threshold, sensitivity, and specificity [19]. Finally, feature importance was calculated for the model combining all fourteen pathologist scores and computer vision-generated cell density.

A *p*-value less than 0.05 was considered statistically significant. Python 3.6 Scikit-Learn 0.24.2 was used for the machine learning analysis, Python Scikit-image 0.17.2 to was used for the computer vision analysis, and Stata version 14.0 was used for descriptive statistics and logistic regression models [20].

## Results

### Patient characteristics

A total of 147 OA patients and 60 RA patients were included in the analysis (Table 1). A greater proportion of RA patients were female (83.3%) compared to OA patients (61.2%) (*p* = 0.002). OA patients had a higher median BMI than RA patients (*p* = 0.006). More RA patients reported a history of cigarette use (60.0%) than OA patients (37.4%) (*p* = 0.003). RA patients had elevated CRP values compared to OA (*p* < 0.001). The median duration since diagnosis was lower in OA patients compared to RA patients (*p* = 0.032). We measured serum RF and CCP by ELISAs on all OA patients and found that no OA patients in our cohort harbored CCP antibodies and only one had RF positivity (2.5 times upper limit of normal) without any signs and symptoms of RA. A total of 50.0% of the RA patients had positive RF, and 78.4% had positive anti-CCP.

**Table 1:** Patient characteristics

Feature	OA *N*=147	RA *N*=60	*p*-value
Age, mean (SD)	65.2 (6.6)	64.0 (9.1)	0.32
Sex: female	90 (61.2%)	50 (83.3%)	**0.002**
BMI, median (IQR)	30.0 (27.1, 3)	28.1 (23.7, 33.2)	**0.006**
Race			0.95
White	117 (79.6%)	46 (76.7%)	
Asian	8 (5.4%)	3 (5.0%)	
Black	15 (10.2%)	6 (10.0%)	
Other^*^	4 (2.7%)	2 (3.3%)	
Missing	3 (2.0%)	3 (5.0%)	
History of cigarette use	55 (37.4%)	36 (60.0%)	**0.003**
ESR (mm/hr), median (IQR)	14 (6.5, 25.5)	14.5 (7.5, 25.5)	0.50
CRP, median (IQR)	0.16 (0.08, 0.36)	1.05 (0.0, 2.4)	**<0.001**
RF positive	1 (0.7%)	30 (50.0%)	
Missing		3 (5.0%)	
Anti-CCP interpretation			
Negative	147 (100.0%)	12 (20.0%)	
Positive: 1–3× ULN		13 (21.7%)	
High positive: >3× ULN		34 (56.7%)	
Missing		1 (1.7%)	
DAS28-ESR, mean (SD)	n/a	3.9 (1.2)	n/a
DAS28-CRP, mean (SD)	n/a	3.8 (1.3)	n/a
Duration since diagnosis, median (IQR)	7.2 (3.2, 15.0)	12.1 (3.7, 19.4)	**0.032**
Duration since symptom onset, median (IQR)	10.6 (5.7, 19.6)	14.9 (4.7, 22.5)	**0.34**
Currently using NSAIDs	85 (57.8%)	32 (53.3%)	
Missing		3 (5.0%)	
Currently using oral steroids	2 (1.4%)	23 (38.3%)	
Missing		1 (1.7%)	
Currently using methotrexate		31 (51.7%)	
Missing	147 (100.0%)	1 (1.7%)	
Currently taking any other DMARD		17 (28.3%)	
Missing	147 (100.0%)	2 (3.3%)	
Currently taking a biologic		33 (55.0%)	
Missing^a^	147 (100.0%)	2 (3.3%)	
Which biologic			
Abatacept (Orencia)		4 (6.7%)	
Adalimumab (Humira)		6 (10.0%)	
Certolizumab (Cimzia)		1 (1.7%)	
Etanercept (Enbrel)		15 (25.0%)	
Infliximab (Remicade)		1 (1.7%)	
Tocilizumab (Actemra)		3 (5.0%)	
Tofacitinib (Xeljanz)		3 (5.0%)	
Missing^a^	147 (100.0%)	27 (45.0%)	

Data represents *N* (%) unless stated otherwise. “Missing” lines indicate the number (and %) of patients for whom data is not available for a given feature. ^a^Data on biologic usage was not collected in OA patients. *OA* osteoarthritis, *RA* rheumatoid arthritis, *SD* standard deviation, *IQR* interquartile range, *DAS28* Disease Activity Score-28, *ESR* erythrocyte sedimentation rate, *mm/hr* millimeters/hour, *CRP* C-reactive protein, *RF* rheumatoid factor, *CCP* cyclic citrullinated peptide, *ULN* upper limit of normal, *NSAIDs* non-steroidal anti-inflammatory drugs, *DMARD* disease-modifying antirheumatic drug. A chi-squared test was performed to assess statistically significant differences between RA and OA patients. Bold represents *p* < 0.05. ^*^Other category of race includes American Indian, Alaskan Native, Native Hawaiian, Pacific Islander, and other race. Duration since diagnosis and duration since symptom onset represent number of years.

### Comparison of OA and RA synovial histologic features

Fibrosis (*p* < 0.001) and mast cell presence (*p* < 0.001) were significantly more common in OA (Table 2). In fact, these two features were almost universally present in OA (95.2% and 99.3%, respectively). There was no statistically significant difference in mucoid change (which was common in both diseases) and germinal centers (which were very rare in both diseases) between patients with OA and RA. To test the hypothesis that fibrosis and mast cells were more commonly observed in OA because there are fewer lymphocytic infiltrates in OA than RA and these features are thus more easily observed, we ran adjusted logistic regression models. Fibrosis (all grades) and mast cells remained statistically significantly associated with the outcome after adjusting for lymphocytic infiltrates in these models. Histologic features of the synovium that were increased in RA compared to OA included lymphocytic inflammation (*p* < 0.001), lining hyperplasia (*p* < 0.001), neutrophils (*p* < 0.001), detritus (*p* < 0.001), plasma cells (*p* < 0.001), Russell bodies (*p* = 0.019), binucleate plasma cells (*p* < 0.001), sub-lining giant cells (*p* < 0.001), synovial lining giant cells (*p* = 0.003), and fibrin (*p* < 0.001) (Table 2). Computer vision quantification of mean cell density per mm^2^ of tissue was significantly lower in patients with OA (2900) compared to those with RA (4196) (*p* < 0.001).

**Table 2:** Synovial histologic features of osteoarthritis vs rheumatoid arthritis

Feature	OA *N*=147	RA *N*=60	*p*-value
Higher in RA			
Lymphocytic inflammation			**<0.001**
None	43 (29.3%)	7 (11.7%)	
Mild (0–1 perivascular aggregates per low power field)	64 (43.5%)	20 (33.3%)	
Moderate (>1 perivascular aggregate + focal interstitial infiltration)	30 (20.4%)	14 (23.3%)	
Marked (both perivascular and widespread interstitial aggregates)	9 (6.1%)	15 (25.0%)	
Band-like	1 (0.7%)	4 (6.7%)	
Lining hyperplasia			**<0.001**
Normal lining	10 (6.8%)	0 (0.0%)	
2–3 cells thick	97 (66.0%)	16 (26.7%)	
3–4 cells thick	38 (25.9%)	26 (43.3%)	
> 4 cells thick	2 (1.4%)	18 (30.0%)	
Neutrophils	1 (0.7%)	13 (21.7%)	**<0.001**
Plasma cells (×25 mag)			**<0.001**
< 10% plasma cells within lymphocytic aggregates	125 (85.0%)	33 (55.0%)	
< 50% plasma cells	18 (12.2%)	17 (28.3%)	
>50% plasma cells	4 (2.7%)	10 (16.7%)	
Binucleate plasma cells	16 (10.9%)	19 (31.7%)	**<0.001**
Russell bodies	13 (8.8%)	13 (21.7%)	**0.019**
Sub-lining giant cells	2 (1.4%)	9 (15.0%)	**<0.001**
Synovial giant cells	25 (17.0%)	22 (36.7%)	**0.003**
Missing	0 (0.0%)	2 (3.3%)	
Fibrin	13 (8.8%)	27 (45.0%)	**<0.001**
Detritus	62 (42.2%)	42 (70.0%)	**<0.001**
Computer vision quantification of cell density, mean	**2900**	**4196**	**<0.001**
Higher in OA			
Fibrosis			**<0.001**
None	7 (4.8%)	40 (66.7%)	
Focal	85 (57.8%)	16 (26.7%)	
Widespread or band-like	55 (37.4%)	4 (6.6%)	
Mast cells	146 (99.3%)	22 (36.7%)	**<0.001**
Missing	0 (0.0%)	1 (1.7%)	
No difference			
Synovial mucoid change			0.73
None	6 (4.1%)	5 (8.3%)	
Slight (perivascular or focal interstitial)	63 (42.9%)	22 (36.7%)	
Moderate (perivascular or focal interstitial)	50 (34.0%)	22 (36.7%)	
Marked (perivascular or focal interstitial)	17 (11.6%)	6 (10.0%)	
Myxomatous	11 (7.5%)	4 (6.7%)	
Missing	0 (0.0%)	1 (1.7%)	
Germinal centers	1 (0.7%)	2 (3.3%)	0.20

Data represents *N* (%). A chi-squared test was performed to assess statistically significant differences between RA and OA patients. Bold represents *p*-value < 0.05

### Supervised machine learning to distinguish OA vs RA

**Fig. 3:**
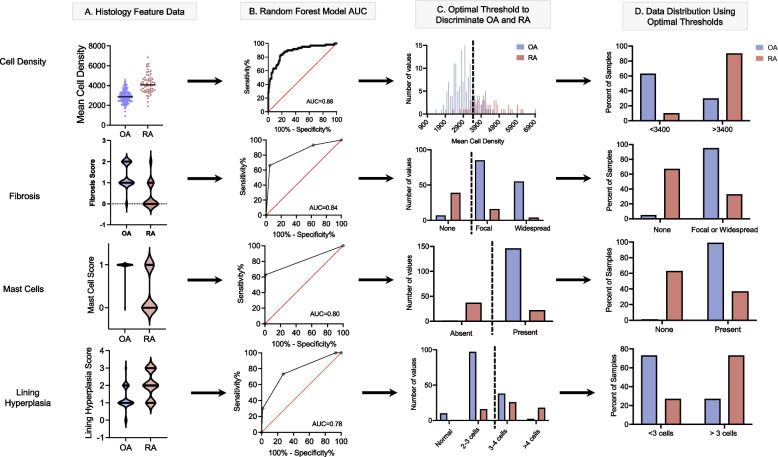
Discovery of optimal thresholds for the top four most predictive histology features to discriminate synovial tissue samples from patients with OA from those with RA. **A** Raw histology feature scores in patients with OA and RA. **B** AUC curves extracted from Random Forest machine learning model. **C** Distribution of raw OA and RA histology feature scores and optimal threshold values extracted from Random Forest machine learning model. **D** Percent of OA and RA samples above or below optimal thresholds identified in C. OA osteoarthritis, RA rheumatoid arthritis, AUC area under the receiver operating characteristic curves

Using disease state OA versus RA as classifiers and histology scores as inputs, we calculated thresholds to optimally distinguish the two disease states for the fourteen pathologist-scored histology features and the computer vision quantification of cell density, and we evaluated the discriminative power of the features according to the area under the curve (AUC) generated from tuning the cutoff threshold (Fig. 3).

Together, the 14 pathologist-scored features yielded a micro-AUC of 0.85±0.06 and macro-AUC of 0.85 for distinguishing OA and RA. By comparison, using computer vision-generated cell density scores alone yielded a similar micro-AUC 0.87 (macro-AUC: 0.88). Finally, combining the computer vision score of cell density with the 14 pathologist scores further improved the micro-AUC to 0.92 ± 0.06 (macro-AUC 0.91). Micro- and macro-precision, recall, and F1 scores, along with the out-of-bag error for each model, are provided in Supplemental Table 1. Feature importance scores for the combined model were calculated and are shown in Table 3: the four most important features that distinguished OA from RA were mast cells followed by cell density, fibrosis, and lining hyperplasia.

**Table 3:** Feature importance, macro area under receiver operating characteristic curves (macro-AUC), and optimal thresholds of the synovial features in distinguishing OA and RA patients

Feature	Feature importance^**a**^	macro-AUC	Optimal threshold OA vs RA^**b**^
Mast cells	0.34	0.80	Present vs none
Automated cell density	0.25	0.88	<3400^c^ cells/mm^2^
Fibrosis	0.11	0.84	Focal and widespread vs none
Lining hyperplasia	0.10	0.78	Normal lining or 2–3 cells thick vs >3–4 cells thick or > 4 cells thick
Fibrin	0.05	0.68	None vs present
Sub-lining giant cells	0.05	0.57	None vs present
Lymphocytic inflammation	0.04	0.69	None and mild (0–1 perivascular aggregates per low power field) vs marked (both perivascular and widespread interstitial aggregates) and band-like
Neutrophils	0.02	0.60	None vs present
Detritus	0.01	0.64	Absent vs present (small or large particles)
Plasma cells	0.01	0.66	<50% plasma cells
Binucleate plasma cells	0.01	0.60	None vs present
Synovial giant cells	0.01	0.58	None vs present
Germinal centers	0.01	0.51	None vs present
Mucoid change	0.00	0.50	No optimal threshold
Russell bodies	0.00	0.56	None vs present

*macro-AUC* macro area under the receiver operating curve

^a^Feature importance scores represent scores for the supervised machine learning model including all fourteen pathology scores and the computer vision-generated cell density

^b^See the Appendix for a full list of categorical variables

^c^Computer vision-quantified cell density measured in mean cells per mm^2^ of tissue

### Thresholds to distinguish OA vs RA

The top four features with the highest individual discriminative power were the computer vision score of cell density (macro-AUC = 0.88), fibrosis (macro-AUC = 0.84), mast cells (macro-AUC = 0.80), and lining hyperplasia (macro-AUC = 0.78) (Table 3). With Youden’s *J* statistic, we discovered that the threshold of cell density lower than 3400 cells per mm^2^ distinguished OA from RA synovium with a sensitivity of 0.82 and specificity of 0.82. The thresholds for the pathologist-scored features for distinguishing OA from RA synovium were the following: focal and widespread fibrosis (vs absence), presence of mast cells (vs absence), and normal or up to 2–3 cells of lining hyperplasia (vs 3–4 or >4 cells) (Fig. 3). Optimal thresholds for the full list of features are provided in Table 3.

## Discussion

Using two well-characterized cohorts of OA and RA patients, we found that H&E-stained images from OA and RA synovial biopsies were distinguishable using 14 pathologist-scored features, computer vision-quantified cell density, or their combination, with AUCs of 0.85, 0.88, and 0.91, respectively. Mast cells and the presence of fibrosis were much more common in OA than in RA synovial biopsies. On the other hand, synovium from patients with RA had increased lining hyperplasia, lymphocytic inflammation, neutrophils, detritus, plasma cells, Russell bodies, binucleate plasma cells, sub-lining giant cells, synovial lining giant cells, and fibrin. The top four features that distinguished OA and RA patients were mast cells, mean cell density, fibrosis, and lining hyperplasia. Finally, we discovered that a threshold of greater than 3400 cells per mm^2^ distinguishes OA from RA synovium with a sensitivity of 0.82 and specificity of 0.82. Thus, automated whole slide cell density can potentially be used as a screening tool in research and clinical settings.

The careful annotations of specific cellular and extracellular features in OA and RA yielded some interesting insights into the two diseases. Lymphocytic inflammation was not uncommon in samples from patients with OA. A total of 27.2% of OA patients had moderate or greater than moderate synovial lymphocytic inflammation, defined as >1 perivascular aggregate per high-power field [7]. In studies of RA, aggregates of lymphocytes have consistently been shown to be associated with increased levels of cytokines, chemokines [21–23], and RA-specific autoantibodies [24, 25] and to be predictive of response to TNF inhibitor [24] and rituximab [26]. However, lymphocyte aggregates are not specific to RA [21]. In this cohort, one-third of patients with OA harbor at least moderate lymphocytic aggregates, underscoring the lack of specificity in our definitions of aggregates. It is possible that lymphocytic infiltrates in our patients with OA may have been caused by undiagnosed concomitant crystalline diseases such as calcium pyrophosphate deposition disease or gout. However, this finding is also in agreement with others who have found that there may be a distinct inflammatory OA subtype [27, 28] that may benefit from different treatment approaches.

Fibrosis and mast cells have previously been reported in OA synovium by other investigators [29–34]. Our study adds to the literature by demonstrating that fibrosis and mast cells are almost always observed in OA (95% and 99%, respectively) and that they are key features that help distinguish RA versus OA. There are two important limitations of this observation. Firstly, these two features were scored as binary, not continuous, and, as can be seen by the angular ROC, this may bias the search for their role and the thresholds in the classification task. Secondly, RA synovial tissue samples were fresh-frozen in optimal cutting temperature compound, and the OA tissues were formalin-fixed and paraffin-embedded. Since paraffin-embedding better preserves morphological details, it is possible and even likely that mast cells were more readily detectable in OA samples, and this difference in sample processing could have contributed to the importance ascribed to mast cells in our analysis. It is less likely, but not impossible, that this difference in sample processing would affect the assessment of fibrosis. Mast cells and fibrosis were inversely associated with other inflammatory features, such as lymphocytes and plasma cells, consistent with prior studies [29, 30].

The finding of increased detritus—small fragments of cartilage or bone—in RA compared to OA was not anticipated, since cartilage damage is a hallmark of OA. One possible explanation is that detritus is increased in RA because intense inflammation is more destructive and may yield larger and therefore more visually obvious debris particles, whereas the cartilage debris generated in response to OA-related damage is smaller and invisible by 10–40× imaging. However, this may also reflect the more advanced damage in the RA joints in patients at the time of arthroplasty.

Several inflammatory features that are typically associated with inflammatory RA such as binucleate plasma cells, Russell bodies, and plasma cells were observed in 11%, 9%, and 15% of OA patients, respectively. This was not anticipated, as plasma cell infiltration of RA synovium has been thought to be related to the fact that patients with RA tend to harbor autoantibodies, such as RF and CCP. Since none of the OA patients in this cohort harbored CCP and only one (0.7%) harbored RF, this finding suggests the non-autoantibody functions of plasma cells in synovial tissue inflammation warrant further exploration.

Neutrophils, which were observed in 22% of RA cases, were very rare (<1%) in OA. We previously observed an association of synovial neutrophils and fibrin, the final product of the clotting cascade, with prolonged morning stiffness in patients with RA [35]. Morning stiffness that lasts for more than 1 h is rare in OA. Thus, our observation that neutrophils are exceedingly rare in OA underscores the possibility that neutrophils together enmeshed in fibrin clots may indeed play a role in the prolonged duration of RA-related morning stiffness. Furthermore, OA stiffness, which is classically either unchanged or worse with activity, likely has a different etiology. Given the well-established contribution of fibrosis to stiffness in other organs [36], it is possible that synovial fibrosis contributes to stiffness in patients with knee OA, as previously proposed [37].

In addition to the above-mentioned sample processing limitation, our study has some other noteworthy limitations. For one, the study population is a convenience sample of OA and RA patients seeking knee arthroplasty at a high-volume, tertiary care hospital in the USA, and thus, the findings may not be applicable to early-stage patients or to joints other than knees. Future studies will compare these histology assessments in other joints and stages of disease. Our sample size is also relatively small, and we did not conduct external validations due to data availability. Further efforts on the evaluation of our model on other independent data sets are needed for justifying its generalizability. We also limited our study to patients who met the classification criteria for OA and RA. While the classification criteria for OA include criteria to help exclude RA, such as less than 30 min of morning stiffness, negative rheumatoid factor, and erythrocyte sedimentation rate less than 40 mm/h, the classification criteria for RA do not include features to help exclude OA and it is likely that many patients with RA also have OA. Though many of the RA patients in our study may have had coincident OA, their synovium was distinguishable from those with OA. We also do not know if these features are distinguishing other causes or types of synovitis, such as psoriatic arthritis and lupus, or if they are better considered methods for distinguishing inflammatory from non-inflammatory pathology. In addition, we only used cell density as an automated computer vision-based feature in our analysis. Identification of additional informative computer vision features for distinguishing OA and RA would warrant further exploration in the future. Finally, 55.0% of the RA patients in this study reported taking a biologic, which would be expected to hinder the ability of the pathologist or our models to discriminate OA from RA since they attenuate inflammation. However, despite the high prevalence of biologic use, we found that the vast majority of samples from patients with RA could be discriminated from those with OA.

Strengths of this study include well-characterized cohorts of OA and RA and an expert musculoskeletal pathologist who has scored and graded the slides for both cohorts. We also demonstrate the utility of cell density, an automated measure by computer vision which can be universally used without an expert pathologist and offers scalability, a quick turn-around, and minimal cost. Previous application of machine learning in rheumatic diseases has involved identifying patients with RA from clinical data, billing codes, and natural language processing-derived concepts in electronic health records [38–40]. Our group has used machine learning to develop algorithms to use synovial histology features to predict gene expression subsets in RA [7] and computer vision algorithms to quantify RA synovial inflammation as measured by cell density [8]. The results presented here extend these studies and indicate that computer vision analysis of standard-of-care pathology slides scanned within electronic health records might also be useful to discriminate patients with RA from those with OA. This has the potential to help clinicians distinguish previously unrecognized or undiagnosed RA who undergo TKR in the future. Presently, we hope this algorithm can help other translational researchers generate more accurate and precise quantification of synovial inflammation for their study comparisons.

In summary, pathologist-scored mast cells, fibrosis, and lining hyperplasia were the most important pathologist-scored features for discriminating OA and RA synovium. A threshold synovial cell density of >3400 yields a sensitivity of 0.82 and a specificity of 0.82 for distinguishing OA from RA. Future efforts will attempt to identify additional informative computer vision features as well as comparisons of their performance on other clinical cohorts.

### Supplementary Information

**Additional file 1: Supplemental Table 1.** Performance metrics for three models in distinguishing OA vs. RA. AUC = area under the receiver operating curve.

## Data Availability

The datasets generated and analyzed during this study are available from the corresponding author on reasonable request.

## Appendix

Human pathologist-scoring system of fourteen features applied to all samples. A portion of these features are illustrated at https://www.hss.edu/pathology-synovitis.asp.

**1. Lymphocytic inflammation:** inflammation consisting of any combination of lymphocytes and plasma cells around blood vessels (perivascular aggregates) or in the interstitium

0: None

1: Mild (0–1 perivascular aggregates per low power field)

2: Moderate (>1 perivascular aggregate + focal interstitial infiltration)

3: Marked (both perivascular and widespread interstitial aggregates)

4: Band-like

**2. Lining hyperplasia:** The number of cells that comprise the thickness of the synovial lining layer. These cells assist in modifying the content of the synovial fluid (make lubricin).

0: Normal lining

1: 2–3 cells thick

2: 3–4 cells thick

3: > 4 cells thick

**3. Neutrophils:** Polymorphonuclear leukocytes. Of note, marginating neutrophils are more likely due to acute stress of surgery than part of a disease phenotype. Therefore, only neutrophils that are present in the interstitium or synovial lining are scored positively.

0: None

1: Present (granulation tissue, interstitial, or marked)

**4. Plasma cells (×25 mag):** Percentage of lymphocytic infiltration, regardless of distribution, that consists of morphologically recognizable plasma cells. Average the percent of all plasma cells over all the inflammation in all fields.

0: < 10% plasma cells within lymphocytic aggregates

1: < 50% plasma cells

2: >50% plasma cells

**5. Binucleated plasma cells:** plasma cells with two or more nuclei

0: None

1: Present

**6. Russell bodies:** plasma cells engorged with bright red substance (antibodies/immunoglobulin)

0: None

1: Present

**7. Sub-lining giant cells:** multinucleated giant cells below the synovial lining layer

0: None

1: Present

**8. Synovial lining giant cells:** multinucleated giant cells in the synovial lining layer

0: None

1: Present

**9. Fibrin:** deposits of fibrinous material or reddish-pink disorganized material on the surface of the synovial membrane

0: None

1: Present

**10. Fibrosis:** Collagen exudate that looks like extremely pink extracellular material

0: None

1: Focal

2: Widespread or band-like

**11. Mast cells:** cells with round or slightly oval cytoplasm with blue granules, and a nucleus that is round and dark, but occasionally oval, centrally placed

0: None

1: Present

**12. Mucoid change:** bluish extracellular material that may appear as something missing from the slide or negative space. Mucin lends the synovial matrix a gelatinous character. It represents sulfated proteoglycans and or non-collagenous proteins

0: None

1: Slight (perivascular or focal interstitial)

2: Moderate (perivascular or focal interstitial)

3: Marked (perivascular or focal interstitial)

4: Myxomatous

**13. Germinal centers:** well-defined collections of enlarged lymphoid cells. These are immunoblasts (small cleaved, small noncleaved, large cleaved, etc.) with prominent nucleoli, tingible body macrophages that contain nuclear material eating nuclear dust, karyorrhexis

0: None

1: Present

**14. Detritus:** bits of bone or cartilage embedded in the synovium

0: Absent

1: Present (small or large particles)

## Abbreviations

OA: Osteoarthritis
RA: Rheumatoid arthritis
H&E: Hematoxylin and eosin
TKR: Total knee replacement
KL: Kellgren-Lawrence
ACR: American College of Rheumatology
BMI: Body mass index
ESR: Erythrocyte sedimentation rate
CRP: C-reactive protein
RF: Rheumatoid factor
CCP: Cyclic citrullinated peptide
STROBE: Strength of Reporting in Observational Studies in Epidemiology
SD: Standard deviation
IQR: Interquartile range
AUC: Area under the receiver operating curve
ROC: Receiver operating characteristic

## References

[1] NIH consensus conference: total hip replacement (1995). NIH Consensus Development Panel on Total Hip Replacement. JAMA.

[2] Slansky E, Li J, Häupl T, Morawietz L, Krenn V, Pessler F (2010). Quantitative determination of the diagnostic accuracy of the synovitis score and its components. Histopathology..

[3] Krenn V, Morawietz L, Burmester GR (2006). Synovitis score: discrimination between chronic low-grade and high-grade synovitis. Histopathology..

[4] Krenn V, Morawietz L, Häupl T, Neidel J, Petersen I, König A (2002). Grading of chronic synovitis--a histopathological grading system for molecular and diagnostic pathology. Pathol Res Pract.

[5] Zhang F, Wei K, Slowikowski K (2019). Defining inflammatory cell states in rheumatoid arthritis joint synovial tissues by integrating single-cell transcriptomics and mass cytometry. Nat Immunol.

[6] Najm A, le Goff B, Venet G (2019). IMSYC immunologic synovitis score: a new score for synovial membrane characterization in inflammatory and non-inflammatory arthritis. Joint Bone Spine.

[7] Orange DE, Agius P, DiCarlo EF (2018). Identification of three rheumatoid arthritis disease subtypes by machine learning integration of synovial histologic features and RNA sequencing data. Arthritis Rheum.

[8] Guan S, Mehta B, Slater D, et al. Rheumatoid arthritis synovial inflammation quantification using computer vision. ACR Open Rheumatol. 10.1002/acr2.11381 Published online January 10, 2022. 10.1002/acr2.11381 PMC8992472 35014221

[9] Altman R, Asch E, Bloch D (1986). Development of criteria for the classification and reporting of osteoarthritis: classification of osteoarthritis of the knee. Arthritis Rheum.

[10] Kellgren JH, Lawrence JS (1957). Radiological assessment of osteo-arthrosis. Ann Rheum Dis.

[11] Goodman SM, Mirza SZ, DiCarlo EF (2020). Rheumatoid arthritis flares after total hip and total knee arthroplasty: outcomes at one year. Arthritis Care Res.

[12] Goodman SM, Bykerk VP, DiCarlo E (2018). Flares in patients with rheumatoid arthritis after total hip and total knee arthroplasty: rates, characteristics, and risk factors. J Rheumatol.

[13] Aletaha D, Neogi T, Silman AJ (2010). 2010 Rheumatoid arthritis classification criteria: an American College of Rheumatology/European League Against Rheumatism collaborative initiative. Arthritis Rheum.

[14] Arnett FC, Edworthy SM, Bloch DA (1988). The American Rheumatism Association 1987 revised criteria for the classification of rheumatoid arthritis. Arthritis Rheum.

[15] von Elm E, Altman DG, Egger M, Pocock SJ, Gøtzsche PC, Vandenbroucke JP. The Strengthening the Reporting of Observational Studies in Epidemiology (STROBE) statement: guidelines for reporting observational studies. J Clin Epidemiol. 2008;61(4). 10.1016/j.jclinepi.2007.11.008. 10.1016/j.jclinepi.2007.11.008 18313558

[16] Otsu N (1979). A threshold selection method from gray-level histograms. IEEE Trans Syst Man Cybern.

[17] Cawley GC, NLCT. (2010). On over-fitting in model selection and subsequent selection bias in performance evaluation. J Mach Learn Res.

[18] Pereira RB, Plastino A, Zadrozny B, Merschmann LHC (2018). Correlation analysis of performance measures for multi-label classification. Inf Process Manag.

[19] Youden WJ (1950). Index for rating diagnostic tests. Cancer..

[20] van der Walt S, Schönberger JL, Nunez-Iglesias J (2014). scikit-image: image processing in Python. PeerJ..

[21] van de Sande MGH, Thurlings RM (2011). Presence of lymphocyte aggregates in the synovium of patients with early arthritis in relationship to diagnosis and outcome: is it a constant feature over time?. Ann Rheum Dis.

[22] Yanni G, Whelan A, Feighery C (1993). Contrasting levels ofin vitrocytokine production by rheumatoid synovial tissues demonstrating different patterns of mononuclear cell infiltration. Clin Exp Immunol.

[23] Manzo A, Paoletti S, Carulli M (2005). Systematic microanatomical analysis of CXCL13 and CCL21in situ production and progressive lymphoid organization in rheumatoid synovitis. Eur J Immunol.

[24] Klaasen R, Thurlings RM, Wijbrandts CA (2009). The relationship between synovial lymphocyte aggregates and the clinical response to infliximab in rheumatoid arthritis: a prospective study. Arthritis Rheum.

[25] Cantaert T, Timmer T, Vandooren B (2007). Synovial T/B cell lymphoid aggregates regulate the production of rheumatoid arthritis-specific autoantibodies. Clin Immunol.

[26] Thurlings RM, Vos K, Wijbrandts CA, Zwinderman AH, Gerlag DM, Tak PP (2008). Synovial tissue response to rituximab: mechanism of action and identification of biomarkers of response. Ann Rheum Dis.

[27] Dell’Isola A, Steultjens M (2018). Classification of patients with knee osteoarthritis in clinical phenotypes: data from the osteoarthritis initiative. PLoS One.

[28] Lv Z, Yang YX, Li J, et al. Molecular classification of knee osteoarthritis. Front Cell Dev Biol 2021;0. 10.3389/fcell.2021.725568 10.3389/fcell.2021.725568 PMC8429960 34513847

[29] Minten MJM, Blom A, Snijders GF (2019). Exploring longitudinal associations of histologically assessed inflammation with symptoms and radiographic damage in knee osteoarthritis: combined results of three prospective cohort studies. Osteoarthr Cartil.

[30] Abdul N, Dixon D, Walker A (2015). Fibrosis is a common outcome following total knee arthroplasty. Sci Rep.

[31] de Lange-Brokaar BJE, Kloppenburg M, Andersen SN (2016). Characterization of synovial mast cells in knee osteoarthritis: association with clinical parameters. Osteoarthr Cartil.

[32] Klein-Wieringa IR, de Lange-Brokaar BJE, Yusuf E (2016). Inflammatory cells in patients with endstage knee osteoarthritis: a comparison between the synovium and the infrapatellar fat pad. J Rheumatol.

[33] Gruber B, Poznansky M, Boss E, Partin J, Gorevic P, Kaplan AP (1986). Characterization and functional studies of rheumatoid synovial mast cells. Activation by secretagogues, anti-IgE, and a histamine-releasing lymphokine. Arthritis Rheum.

[34] Pu J, Nishida K, Inoue H, Asahara H, Ohtsuka A, Murakami T (1998). Mast cells in osteoarthritic and rheumatoid arthritic synovial tissues of the human knee. Acta Med Okayama.

[35] Orange DE, Blachere NE, DiCarlo EF (2020). Rheumatoid arthritis morning stiffness is associated with synovial fibrin and neutrophils. Arthritis Rheum.

[36] Rockey DC, Bell PD, Hill JA (2015). Fibrosis--a common pathway to organ injury and failure. N Engl J Med.

[37] Kuo SJ, Yang WH, Liu SC, Tsai CH, Hsu HC, Tang CH (2017). Transforming growth factor β1 enhances heme oxygenase 1 expression in human synovial fibroblasts by inhibiting microRNA 519b synthesis. PLoS One.

[38] Jiang M, Li Y, Jiang C, Zhao L, Zhang X, Lipsky PE (2021). Machine learning in rheumatic diseases. Clin Rev Allergy Immunol.

[39] Zhou SM, Fernandez-Gutierrez F, Kennedy J (2016). Defining disease phenotypes in primary care electronic health records by a machine learning approach: a case study in identifying rheumatoid arthritis. PLoS One.

[40] Carroll RJ, Eyler AE, Denny JC (2011). Naïve Electronic Health Record phenotype identification for Rheumatoid arthritis. AMIA Annu Symp Proc.

