# Supplementary material for: Machine learning identification of thresholds to discriminate osteoarthritis and rheumatoid arthritis synovial inflammation
Source: Arthritis Res Ther. 2023 Mar 2;25:31. doi: 10.1186/s13075-023-03008-8 (PMC9979511; doi:10.1186/s13075-023-03008-8)
Supplement: Supplementary file 1 — Additional file 1: Supplemental Table 1. Performance metrics for three models in distinguishing OA vs. RA. AUC = area under the receiver operating curve. [file 13075_2023_3008_MOESM1_ESM.pdf]

Supplemental Table 1. Performance metrics for three models in distinguishing OA vs. RA

	**Model 1: Pathologist-Scored Features**	**Model 2: Cell Density Alone**	**Model 3: Pathology-Scored Features + Cell Density**
Micro-AUC (±SD)	0.85±0.06	0.87	0.92±0.06
Macro-AUC	0.85	0.88	0.91
Micro-Precision (±SD)	0.82±0.10	0.91±0.14	0.93±0.08
Macro-Precision	0.89	0.89	0.92
Micro-Recall (±SD)	0.86±0.11	0.84±0.10	0.81±0.09
Macro-Recall	0.8	0.84	0.9
Micro-F1 score (±SD)	0.84±0.10	0.87±0.05	0.87±0.06
Macro-F1 score	0.83	0.86	0.91
Out-of-bag-error (±SD)	0.12±0.01	---	0.09±0.02

AUC = area under the receiver operating curve
